# Treating hidradenitis suppurativa patients with adalimumab: a real-life experience of a tertiary care center in Lisboa, Portugal^⋆^

**DOI:** 10.1016/j.abd.2021.12.004

**Published:** 2022-09-10

**Authors:** José Miguel Neves, Nélia Cunha, André Lencastre, Joana Cabete

**Affiliations:** Department of Dermatology and Venereology, Hospital de Santo António dos Capuchos, Centro Hospitalar Universitário de Lisboa Central, Lisbon, Portugal

Dear Editor,

Hidradenitis Suppurativa (HS) is a debilitating, potentially mutilating, chronic, inflammatory systemic skin disease.^1^, ^2^, ^3^ A long delay between HS onset and its diagnosis is common,^1^, ^4^, ^5^ and it appears to have an impact in response to biological treatment.^5^ Currently, adalimumab is the sole biological approved for the treatment of moderate-to-severe HS.

We conducted a retrospective study to analyze HS patients treated with adalimumab at a terciary health care center in Lisboa, between 2016 and 2019. Epidemiological, clinical, and therapeutic information was retrieved. HS activity and response to adalimumab were monitored at baseline and Weeks 16 (W16), 24 (W24), and 52 (W52). A baseline observation at the clinic and a minimum of 16 weeks of follow-up were required for inclusion. Patients on adalimumab increased doses and cases of paradoxical HS were excluded. Patients could have adjuvant medical treatments when considered suitable. Severity assessment tools were employed, namely Hurley Staging System, International Hidradenitis Suppurativa Severity Score System (iHS4), Dermatology Life Quality Index (DLQI), and visual analog scale for pain (VAS pain). Response to treatment was evaluated using Hidradenitis Suppurativa Clinical Response (HISCR).

Analysis was performed using IBM SPSS Version 24. Independent samples *t*-Test was used to test differences between continuous and categorical variables at a significance level of 0.05. Fischer’s exact test was used to test differences between 2 categorical variables at a significance level of 0.05, two-tailed.

Out of 198 HS patients, 51 started treatments with a biological agent and, of these, 36 were on adalimumab and met the study criteria. The comparison between these 36 patients under adalimumab and the 147 patients without biological treatment can be found in Table 1. At baseline, the severity was significantly higher in the adalimumab group, using both objective (Hurley: 2.6 vs. 1.7; p < 0.001; iHS4: 16.7 vs. 4.1; p < 0.001) and subjective criteria (DLQI: 15.4 vs. 10.4, p = 0.002). Most patients on adalimumab presented with severe disease (iHS4 > 10: 75%; n = 27; iHS4 ≤ 10: 25%; n = 9), 58.3% (n = 21) with Hurley III and 41.7% (n = 15) with Hurley II.

**Table 1:** Epidemiological and clinical data comparing adalimumab and non-biological HS patients.

Features	Adalimumab patients (n = 36)	Non biological (n = 147)	Statistical significance
Fermale prevalence, n (%)	25 (69.4%)	101 (68.7%)	0.981
HS onset (years)^a^	27.7	24.7	0.192
Diagnosis delay (years)^a^	8.0	10.8	0.151
Number of comorbidities^a^	3.8	3.4	0.206
Number of affected sites^a^	3.5	2.7	0.003
BMI^a^	29.3	28.3	0.350
Past/present smoking history, n (%)	26 (72.2%)	95 (64.6%)	0.558
Initial iHS4^a^	16.7	4.1	<0.001
Hurley stage^a^	2.6	1.7	<0.001
Initial DLQI^a^	15.4	10.4	0.001

BMI, Body Mass Index; HS, Hidradenitis Suppurativa.

a Data are reported as means.

At W16, HISCR was achieved in 27 patients (75%). This percentage was similar in patients with moderate (89%, n = 8) and severe disease (70.4%, n = 19) (p > 0.05), The mean iHS4 was reduced from 16.7 to 7.2 (p < 0.001).The time from HS onset to diagnosis was similar (6.7 years vs. 11.8; p = 0.282). At W24 the iHS4 reduction was significant (mean iHS4 = 6.5; (p < 0.001). At W52, the mean iHS4 was 4.7 (p < 0.001), and HISCR was still achieved in 76.7% (n = 23/30) of the patients. Between baseline and W52, DLQI and VAS pain shifted from a mean value of 15.4 to 10.5 (p = 0.001) and 4.4 to 1.8 (p < 0.001), respectively.

Within the first 16 weeks, in order to successfully control HS inflammatory activity, adjuvant, transitory, and medical treatments were employed in 72% (n = 26) of the cases. During the remaining period, adjuvant therapeutics were needed in 20 patients to control episodic flares.

Addressing the differences between patients staged as Hurley II and III (Table 2), 82% (n = 9) of male patients were classified as Hurley III, compared to 48% (n = 12) of females who presented such severity (p = 0.044). At baseline, Hurley II patients presented similar severity (mean iHS4: 13.7 vs. 18.8; p = 0.085) but with a significantlower number of draining fistulae (2.0 vs. 3.9; p = 0.002). While both groups recorded HS improvement under treatment, significantly better control of disease activity at W52 was observed within Hurley II patients (iHS4: 1.3 vs. 7; p = 0.003). Otherwise, the response to adalimumab measured by HISCR achievement at W16 and W52 was superior and similar in Hurley II patients (W16: 93% vs. 61.9%, p = 0.051; W52: 83.3% vs. 72.2%; p = 0.669), respectively.

**Table 2:** Clinical characterization and therapeutic response of Hurley II and Hurley III HS groups.

	Hurley II	Hurley III	Statistical significance (p<0.05)
Number of affected sites	3.1	3.9	0.121
HS onset (years)^a^	29.1	26.7	0.595
Diagnosis delay (years)^a^	6.7	8.9	0.508
Initial iHS4 (n=36)^a^	13.7	18.9	0.085
Initial number of draining fistulas (n=36)^a^	2.0	3.9	0.002
W16 iHS4 (n=36)^a^	3.8	9.6	0.038
W24 iHS4 (n=33)^a^, ^b^	1.2	10.4	<0.001
W52 iHS4 (n=30)^a^, ^c^	1.3	7	0.003
W52 number of draining fistulas (n=30)^a^, ^c^	0.1	1.9	0.002
W16 HISCR (n=36)^a^	93.3% (n=14)	61.9% (n=13)	0.051
W52 HISCR (n=30)^c^	83.3% (n=10)	72.2% (n=13)	0.669
W52 Adalimumab only (n=30)	41.7% (n=5)	27.8% (n=5)	0.461

a Data are reported as means.

b n=33 ‒ two patients were evaluated only at W16 and one had adalimumab discontinued due to primary failure.

c n=30 – two patients had their last evaluation at W24 and one had adalimumab discontinued due to secondary failure.

Considering the last clinical evaluation of all patients, 78% (n = 28) witnessed a reduction of at least 50% of their iHS4 at baseline (p < 0.001). Within the group that did not achieve such a response (n = 8), half presented with more than five draining fistulas at baseline and two of them switched biological treatment.

Clinical trials have shown that adalimumab is an effective treatment for moderate-to-severe HS with inadequate response to conventional treatments,^6^, ^7^, ^8^ with HISCR achievement rates ranging from 40%‒60% in monotherapy.^4^, ^6^, ^8^, ^9^ The present study’s results showed superiority in terms of HISCR achievement at W12/16, W24 and W52 when compared to PIONEER I and II clinical trials and to Marzano et al. multicentre study.^4^, ^6^, ^7^, ^8^ We associated the better results (75% HISCR achievers at W16) with the use of adjuvant intralesional and systemic therapeutics. This, we believe, may be a necessary practice in a real-life setting in order to further reduce inflammation and pain in notably severe cases along with adalimumab induction. Additionally, as flares can still be observed in patients on adalimumab monotherapy, adjuvant therapies may be required to treatment optimization.

The present results showed a greater response to adalimumab in Hurley II patients when compared to Hurley III, especially observable in the mean iHS4 reduction. Also, the delay to HS diagnosis was higher in the Hurley III group. These findings follow the trend within the “Window of Opportunity” hypothesis, which has postulated an inverse relationship between HS duration and/or diagnostic delay and adalimumab effectiveness.^4^, ^9^, ^10^ It has been suggested that starting adalimumab earlier, when HS is characterized by reversible lesions, encompasses the potential to prevent disease progression, development of fistulas, and permanent deformities.^4^, ^10^ Hurley III patients enclose a more severe clinical status, which may justify lower effectiveness of adalimumab. The present findings further highlight the importance of precocious diagnosis, in order to effectively treat and prevent HS natural evolution.

In conclusion, adalimumab is a useful and effective treatment for HS although in monotherapy may not be sufficient to allow optimal control in some patients. This study supports the need for a proactive treatment, underlining the importance of early referral, the precocious use of adalimumab, and of the benefit of adjuvant therapies in patients under adalimumab. We highlight that real-life evidence is still scarce and more studies must be performed to allow more suitable evidence-based therapeutic guidelines.

## Financial support

The authors received no financial support for the research, authorship, and/or publication of this article.

## Authors’ contributions

José Miguel Neves: Approval of the final version of the manuscript; critical literature review; data collection, analysis, and interpretation; effective participation in research orientation; intellectual participation in propaedeutic and/or therapeutic management of studied cases; manuscript critical review; preparation and writing of the manuscript; statistical analysis; study conception and planning.

Nélia Cunha: Approval of the final version of the manuscript; critical literature review; data collection, analysis, and interpretation; effective participation in research orientation; intellectual participation in propaedeutic and/or therapeutic management of studied cases; manuscript critical review; preparation and writing of the manuscript; statistical analysis; study conception and planning.

André Lencastre: Approval of the final version of the manuscript; critical literature review; data collection, analysis, and interpretation; effective participation in research orientation; intellectual participation in propaedeutic and/or therapeutic management of studied cases; manuscript critical review; preparation and writing of the manuscript; statistical analysis; study conception and planning.

Joana Cabete: Approval of the final version of the manuscript; critical literature review; data collection, analysis, and interpretation; effective participation in research orientation; intellectual participation in propaedeutic and/or therapeutic management of studied cases; manuscript critical review; preparation and writing of the manuscript; statistical analysis; study conception and planning.

## Conflicts of interest

The authors declared no potential conflicts of interest with respect to the research, authorship, and/or publication of this article.
